# Beyond the green myth: a scoping review of spatial justice and structural barriers to therapeutic urban agriculture for vulnerable communities

**DOI:** 10.3389/fpubh.2026.1907463

**Published:** 2026-09-29

**Authors:** Xiaoyan Ye, Peili Lu, Qiang Chen

**Affiliations:** 1 Cai Yuanpei School of Art and Design, Shaoxing University, Shaoxing, Zhejiang, China; 2 School of Foreign Languages, Shaoxing University, Shaoxing, Zhejiang, China; 3 Department of Collection Development, Library, Yunnan University, Kunming, Yunnan, China

**Keywords:** eco-gentrification, healing justice, scoping review, spatial justice, therapeutic landscape, urban agriculture, vulnerable populations

## Abstract

**Introduction:**

Motivated by the urgent need to address urban health inequities, this scoping review critiques the mainstream public health discourse that champions therapeutic urban agriculture (UA) as an accessible mental health intervention. This narrative often obscures the systemic spatial inequities and structural barriers marginalized populations face. To address this gap, we systematically mapped the physical, economic, and institutional mechanisms impeding therapeutic UA for vulnerable communities.

**Methods:**

Adhering to JBI and PRISMA-ScR methodologies, we executed a systematic retrieval across Web of Science and Scopus from database inception to March 2026, yielding 304 records. Focusing on English-language empirical literature, the epistemic scope of this synthesis primarily reflects the socio-spatial dynamics and planning frameworks of the Global North, rather than claiming universal global applicability. By applying a Population, Concept, and Context (PCC) framework, we retained and analyzed 30 core empirical articles.

**Results:**

Our thematic synthesis reveals three primary structural barriers constraining UA’s therapeutic potential. Physically, land tenure precarity frequently relegates growers to fragmented or marginalized geographies. Economically, the reliance on competitive short-term funding burdens grassroots initiatives, while successful greening interventions paradoxically trigger eco-gentrification. Institutionally, procedural exclusion and social stigmatization downgrade community-led agricultural spaces to transient interim uses within broader urban agendas.

**Discussion:**

Consequently, the synthesis indicates that implementing localized greening projects without resolving these structural vulnerabilities remains counterproductive. As its primary contribution, this review proposes a multidimensional “healing justice” framework, asserting that to transition UA into a permanent public health infrastructure, urban planning must prioritize structural interventions, such as Community Land Trusts (CLTs) and anti-displacement spatial policies, to secure spatial agency for marginalized groups.

## 1 Introduction

The post-pandemic era has accelerated the reliance on therapeutic urban agriculture (UA) as a public health intervention (1). Mainstream urban planning and public health discourses frequently champion community gardens and urban farms as low-barrier approaches to alleviating mental health disorders and rebuilding social cohesion (2, 3). This optimistic narrative, however, risks perpetuating a depoliticized green myth (4). Recent scholarship in spatial health explicitly cautions that reducing community agriculture to a simplified green prescription undermines the socio-spatial complexity required to sustain these environments (5). The prevailing discourse assumes that the physical presence of urban vegetation inherently distributes equitable therapeutic benefits, largely overlooking the socio-economic drivers dictating green space distribution (6). Consequently, for marginalized groups, the healing potential of these agricultural spaces remains constrained by contested spatial politics and entrenched structural exclusion (7–9).

Accessing therapeutic nature requires vulnerable populations to navigate significant structural impediments. While UA functions as a spatial tactic for cultural resettlement and trauma recovery—particularly for transnational immigrants and refugees (10, 11)—contemporary urban policies rarely accommodate the structural transformations necessary to sustain these practices. Vulnerable communities frequently cultivate on marginalized or remediated lands, operating under the persistent threat of precarious land tenure and speculative development (12–14). Furthermore, successful greening interventions can inadvertently catalyze eco-gentrification, intensifying financial pressures and risking socio-cultural displacement (15, 16). Beyond physical precarity, institutional barriers, including competitive grant cycles and inflexible funding structures, exhaust grassroots organizers and minority-led initiatives (17, 18). Cultivating the land thus transitions from a leisure activity into a necessary practice of reclaiming spatial agency and the right to the city (19, 20).

Despite the growing body of literature on urban gardening and public health (21), a synthesis mapping these intersecting structural barriers remains acutely scarce. While recent systematic reviews robustly validate the clinical efficacy of horticultural therapy in alleviating specific mental health disorders (22), they predominantly operate within a biomedical paradigm. Similarly, scoping reviews focusing exclusively on agronomic dimensions or macro-level food security (23) often overlook the micro-politics of spatial access and structural exclusion. This creates a critical evidence gap. We argue that divorcing clinical outcomes from the spatial realities of land insecurity and procedural exclusion limits the translation of evidence into effective policy, particularly given the critical role of stakeholder engagement in nature-based solutions (24). Consequently, within this review, the concept of “therapeutic” extends beyond physiological metrics to encompass the foundational structural and policy prerequisites necessary for marginalized populations to secure spatial agency and community resilience. Without synthesizing how these systemic hurdles intertwine, urban policy cannot transition UA from a localized practice into a permanent tool for inclusive public health infrastructure (13).

To deconstruct this green myth and guide future policy interventions, we systematically map the intersection of therapeutic UA, spatial justice, and marginalized populations. However, it is imperative to define the epistemic scope of this review. By focusing on English-language empirical literature indexed in major international databases, our synthesis inherently predominantly reflects the structural conditions and theoretical frameworks of the Global North. Therefore, rather than claiming universal global applicability, this study aims to dissect the specific structural typologies of exclusion that often accompany therapeutic greening within these contexts. Utilizing the PCC (Population, Concept, Context) framework and guided by the PRISMA-ScR guidelines (25), our scoping review addresses the following core research questions:

*RQ1*: What are the spatiotemporal distribution and methodological characteristics of the existing empirical literature?

*RQ2*: How do spatial, economic, and institutional barriers interact to impede the therapeutic efficacy of UA interventions for marginalized groups?

*RQ3*: Based on the synthesized evidence, what conceptual framework emerges from the literature to transition therapeutic UA toward spatially just public health infrastructures?

## 2 Materials and methods

We conceptualized and executed this scoping review in accordance with the Joanna Briggs Institute (JBI) methodology (26) and the PRISMA-ScR (Preferred Reporting Items for Systematic reviews and Meta-Analyses extension for Scoping Reviews) framework (25). Appendix A details our methodological compliance and provides the completed 22-item checklist.

### 2.1 Information sources and search strategy

To ensure methodological transparency and capture the historical evolution of spatial policy, we conducted a comprehensive literature retrieval across two electronic databases—Web of Science (Core Collection) and Scopus—searching for peer-reviewed records from database inception up to March 18, 2026. These specific databases were selected for their robust coverage of interdisciplinary urban planning, environmental policy, and social science literature. We constructed the search strategy utilizing a tripartite framework capturing the intervention (therapeutic UA), the context of inequity (spatial justice and institutional barriers), and psychosocial outcomes (mental health and wellbeing). Appendix A (Table A1) documents the exact Boolean search syntax, applied truncation parameters, and specific yield counts for each database.

### 2.2 Eligibility criteria (the PCC framework)

Aligned with JBI guidelines, we defined eligibility utilizing the Population, Concept, and Context (PCC) framework:

*Population*: Studies focusing on marginalized urban populations (e.g., low-income residents, racial minorities, transnational refugees) experiencing systemic spatial and institutional inequities.
*Concept*: Empirical investigations explicitly analyzing physical, economic, or policy barriers to therapeutic UA. We excluded studies focused solely on agronomic yields or clinical horticultural trials devoid of socio-spatial dimensions.
*Context*: Urban environments globally.

We included only peer-reviewed journal articles published in English, excluding non-peer-reviewed gray literature, editorials, and isolated book chapters to maintain empirical rigor.

### 2.3 Study selection process

The initial retrieval yielded 304 records. After removing 55 duplicates via Zotero, 249 unique articles advanced to the primary screening phase. Utilizing the Rayyan web application (27), two independent reviewers (X.Y. and P.L.) screened titles and abstracts against the predefined PCC criteria in a double-blind manner, resolving initial discrepancies through discussion. This stage refined the pool to 73 potentially relevant articles.

Subsequently, the two independent reviewers (X.Y. and P.L.) conducted full-text evaluations. We resolved persisting ambiguities regarding article eligibility through collective deliberation, with a third external collaborator (Q.C.) acting as an independent arbitrator to ensure conceptual fidelity to the PCC framework. Full-text screening eliminated 43 studies lacking empirical analyses of socio-spatial barriers. Appendix C (Table C1) provides a representative matrix of these excluded records alongside their methodological rationales. Ultimately, 30 core articles met all inclusion criteria and advanced to data extraction (Figure 1).

**Figure 1:**
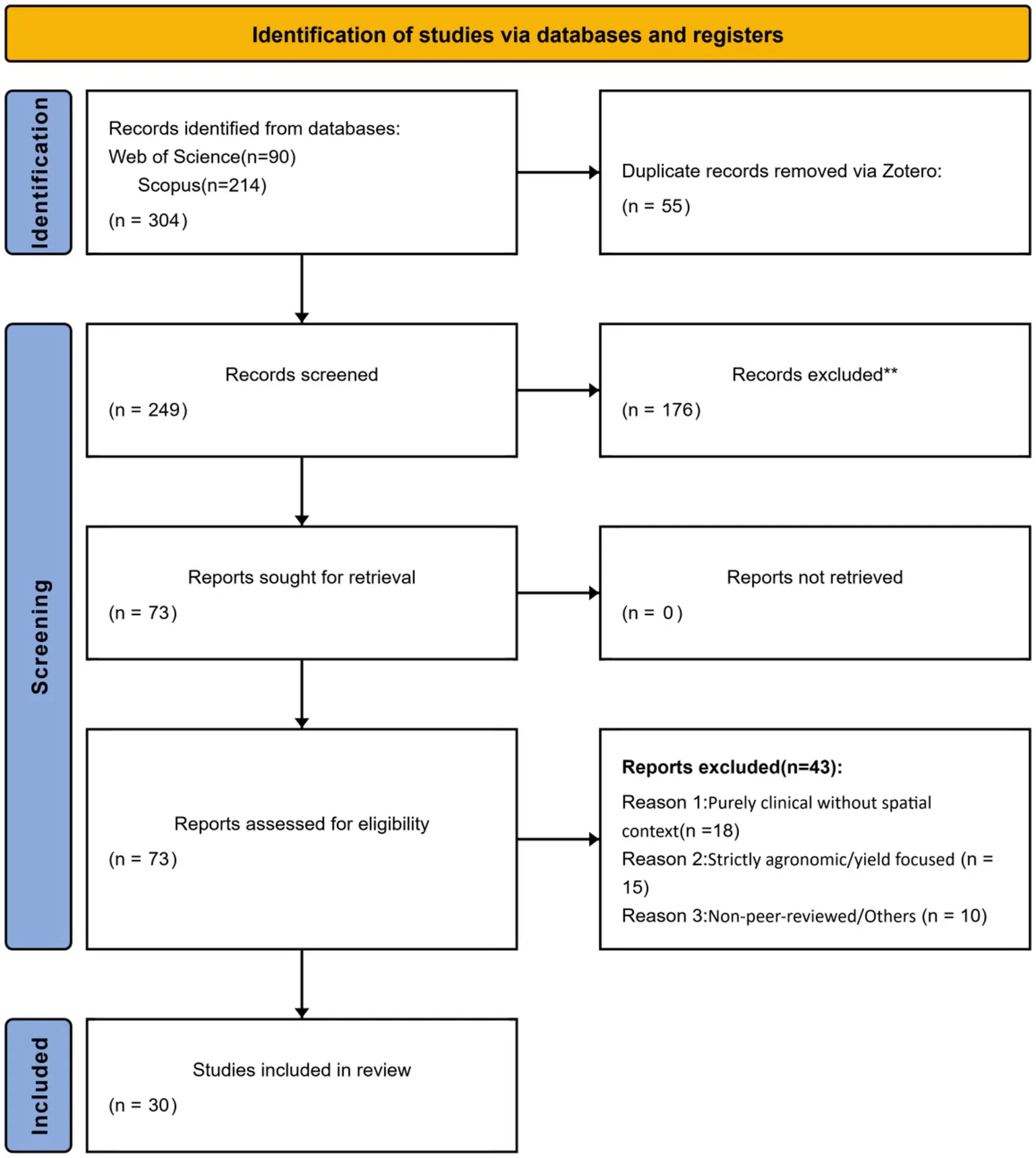
PRISMA flow diagram detailing the study selection process.

### 2.4 Data charting process

To map the evidence landscape, we iteratively pilot-tested a standardized charting form in Microsoft Excel, adhering to established scoping review protocols (28). One reviewer (P.L.) initially executed a 12-dimension charting process to deconstruct the 30 included articles. The primary investigator (X.Y.) subsequently cross-verified the extracted data for accuracy and completeness. The comprehensive evidence matrix in Appendix B (Table B1) documents the specific methodological contexts, barrier manifestations, proposed policy interventions, and primary qualitative quotes for each study.

### 2.5 Synthesis of results

Consistent with PRISMA-ScR guidelines, we omitted a formal risk of bias assessment, as our objective is to map the structural evidence landscape rather than synthesize clinical effect sizes. We deployed a mixed-methods synthesis approach, generating descriptive statistics and geographical heatmaps to illustrate temporal and spatial publication trajectories.

Subsequently, the first author (X.Y.) conducted a Reflexive Thematic Analysis (29, 30) on the extracted qualitative data, which the co-authors critically reviewed. Appendix C (Table C2) details the analytical codebook mapping the conceptual transition from raw empirical indicators to the overarching triad of physical, economic, and institutional barriers. Finally, we integrated visual mapping techniques (e.g., Sankey diagrams) to illustrate the intersecting network between marginalized typologies and structural exclusionary mechanisms.

## 3 Results

### 3.1 Study characteristics and descriptive mapping

All 30 included core studies were published between 2013 and 2025, exhibiting a notable temporal concentration with over three-quarters (*n* = 23) emerging from 2020 onwards (Figure 2a). As indicated by Bailey and Kingsley (31) and Wardle et al. (1), this recent surge reflects a documented academic consensus within the literature on how the global pandemic exacerbated structural vulnerabilities. Within this contemporary evidence base, UA transitions from being framed as a supplementary green amenity to a critical mechanism for urban survival and spatial reclamation.

**Figure 2:**
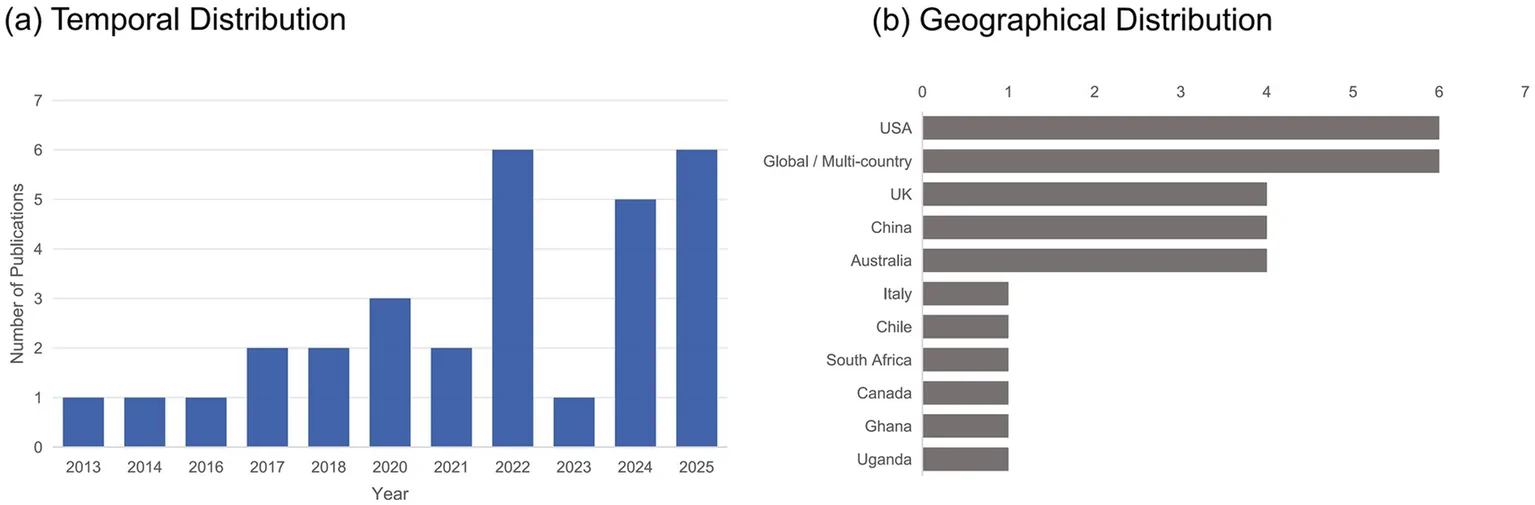
Temporal **(a)** and geographical **(b)** distribution of the 30 included core studies. The post-2020 publication surge **(a)** aligns with increased academic attention to pandemic-exacerbated structural inequities. The geographical mapping **(b)** demonstrates a pronounced concentration in the Global North, contrasted with emerging but fragmented evidence from the Global South (e.g., China, South Africa).

Geographically, the literature demonstrates a pronounced asymmetry, predominantly concentrating in the Global North (Figure 2b). Over half of the empirical studies originate from the United States, the United Kingdom, and Australia. Within these specific political economies, researchers frequently document the green gentrification paradox, wherein large-scale greening interventions inadvertently displace the marginalized residents they ostensibly serve (9, 32). Conversely, empirical evidence from the Global South remains fragmented. Emerging studies from South African informal settlements and densified Chinese megacities reveal a different structural barrier: state-sanctioned urban densification and fragmented governance structures systematically exclude the urban poor from therapeutic landscapes (6, 33, 34). This geographic disparity highlights a critical knowledge gap in the current literature regarding how spatial injustice mutates across diverse political economies.

The synthesis reveals intersecting typologies of marginalization among vulnerable populations. Economically deprived cohorts—including working-class allotment holders and social housing tenants—consistently experience severe spatial precarity. Cultivating in physically constrained or marginal spaces, such as back alleyways and parking lots, these groups operate under continuous eviction threats driven by real estate speculation (35, 36). Simultaneously, racially and ethnically minoritized populations, such as Black urban farmers and Native American communities, encounter systemic barriers rooted in historical land dispossession and the prohibitive costs of remediating toxic industrial legacies (17, 37, 38). Finally, displaced populations, including refugees and transnational migrants, face pronounced procedural exclusion. Their access to landscape justice is severely impeded by linguistic challenges, restrictive seed import policies, and exclusionary local governance contexts (39, 40).

### 3.2 Thematic mapping: the triad of barriers to therapeutic urban agriculture

The thematic mapping demonstrates that the exclusion of vulnerable populations from therapeutic UA is rarely a byproduct of individual disinterest or lack of horticultural skill; rather, the literature frames it as the structural consequence of three intersecting barriers: physical, economic, and institutional. While these barriers operate universally, the synthesis reveals they mutate across localized socio-political contexts. For instance, documented physical exclusion ranges from the extreme territorial fragmentation of peri-urban Italy (13) to the industrial legacies and heavy metal-contaminated soils confronting Black and Brown farmers in Boston (38). Economically, the extracted evidence indicates that financial constraints routinely force grassroots initiatives into a cycle of short-term grant dependency. This precarity inadvertently triggers mission creep, compelling organizations to alter their original community-healing ethos merely to satisfy stringent funding metrics (1, 18). Institutionally, the reviewed studies highlight that therapeutic greening often reproduces systemic inequities. This dynamic marginalizes minority-led farms and reflects entrenched environmental racism and structural exclusion within municipal planning discourse (17, 41). Table 1 synthesizes this landscape of structural inequity, categorizing the triad of barriers across diverse geographies. Appendix B provides the comprehensive evidentiary foundation substantiating these categorizations.

**Table 1:** Landscape of structural inequity: included studies, the triad of barriers, and their synthesized mechanisms in therapeutic UA (*n* = 30).

Study	Location	Target vulnerable population	Physical and spatial barriers	Economic and financial barriers	Institutional and social barriers	Synthesis: link to the triad
Allen and Apsan Frediani (2013) (52)	Ghana	Indigenous farmers, urban poor	Real-estate encroachment; Displacement to peripheries	Cycle of corporate dependency	Stigmatization as squatters; Devaluation of local knowledge	Spatial displacement and corporate economic dependency reinforce the social stigmatization of urban poor populations.
Bailey and Kingsley (2022) (31)	Australia	Migrants, workers	Land tenure insecurity	Lack of material sourcing	Marginalization of UA; Pro-sports municipal bias	Institutional biases prioritize sports over UA, exacerbating land insecurity and material sourcing challenges for migrants.
Brown et al. (2020) (37)	USA	Native Americans (AI/NA)	Poor soil quality (rocks/clay); Irrigation deficits	Poverty traps vs. cheap processed food subsidies	Generational divides; Negative federal food policies	Federal food policies and poverty traps compound physical constraints like poor soil, deepening generational divides.
Contesse et al. (2018) (44)	Chile	Low-income residents	Relegation to disused/marginal spaces	Municipal budget constraints	Fragmented governance; Denied public space status	Fragmented governance and municipal budget constraints directly relegate low-income residents to disused, marginal spaces.
Egoz and De Nardi (2017) (39)	Global / EU	Refugees, migrants	Poor facility provision; Distant locations	Public transport dependence	Racial discrimination; White landscape ideals	Racial discrimination and exclusionary landscape ideals force marginalized groups into distant locations reliant on public transport.
Fanfani et al. (2024) (13)	Italy	Local residents	Territorial fragmentation; Poor accessibility	Unviable without public subsidy	Bureaucratic silos; Administrative path dependence	Bureaucratic silos and reliance on public subsidies exacerbate territorial fragmentation and poor spatial accessibility.
Ferreira et al. (2020) (24)	Global	Low-income, urban poor	Lack of spatial technical support	Financial constraints; Unaffordable time costs	Bureaucratic red tape; Low social cohesion	Bureaucratic red tape and low social cohesion compound the lack of spatial support and unaffordable financial/time costs.
Forbes (2024) (17)	USA	Black urban farmers	Loss of vacant land; Speculative reinvestment	Structural lack of capital	Racial capitalism; White supremacy in UA discourse	Racial capitalism and biased UA discourse drive speculative reinvestment, causing land loss and capital deficits for Black farmers.
Furlong et al. (2025) (46)	UK	Working-class, low-income	High travel distances; Physical isolation via hedges	Rising cost of living	Gang-related safety issues; Social surveillance	Rising living costs and social surveillance create physical isolation and safety issues for working-class populations.
Kenner et al. (2022) (41)	USA	Black and Brown communities	Rapid real estate turnover	Unaffordable long-term maintenance	Performative greening; Disciplinary surveillance	Performative greening and disciplinary surveillance accelerate rapid real estate turnover and unaffordable site maintenance.
Kronsted (2024) (45)	Uganda	Slum youth	Hyper-density; Flood and landslide risks	Disproportionate labor-to-yield costs	Deprivation of political rights; Exclusion from planning	Deprivation of political rights forces slum youth into risk-prone, hyper-dense areas with disproportionate labor-to-yield costs.
Langerman et al. (2025) (10)	Global	Refugees, immigrants	Lack of infrastructure	Unequal funding scales	Procedural exclusion; Passive recipient status	Procedural exclusion and unequal funding relegate refugees to a passive recipient status lacking basic infrastructure.
Li and Huo (2025) (33)	China	Community residents	Complex site conditions; High commuting costs	Long investment cycles; Low economic returns	Conflicting stakeholder interests	Conflicting stakeholder interests and long investment cycles complicate spatial site conditions and increase commuting costs.
Lu et al. (2025) (48)	China	Tenants, youth volunteers	Collective land disputes; Legal grey areas	High infrastructure costs	Rigid local institutions; Suppression of dissent	Rigid local institutions and suppression of dissent create legal grey areas and drive up infrastructure costs for tenants.
Marsh et al. (2020) (47)	Australia	Students, local public	Hostile microclimates; Lack of windbreaks	Overreliance on unpaid volunteer labor	Institutional disconnect; Low community trust	Institutional disconnects and lack of community trust lead to an overreliance on unpaid labor in hostile microclimates.
Otoadese et al. (2025) (40)	Canada	Black African Diaspora	Severe climatic constraints	Multiple minimum-wage employment	Systemic racism; Restrictive seed import policies	Systemic racism and restrictive policies exacerbate severe climatic constraints and the financial burden of multiple low-wage jobs.
Philpott et al. (2023) (42)	USA	Urban gardeners	Insecure land ownership; Small plot sizes	Economic pressures of food security	Sense of temporariness; Tenure instability	Institutional tenure instability and economic pressures of food security result in insecure land ownership and restricted plot sizes.
Rao et al. (2022) (51)	Global	Urban poor, minorities	Expropriation threats	Exploitation of volunteer labor	Devaluation as unreal agriculture; Procedural exclusion	Procedural exclusion and the devaluation of UA legitimize expropriation threats and the economic exploitation of volunteer labor.
Raskin et al. (2021) (35)	USA	Historically underserved	Refusal of secure tenure	Lack of bridging capital	Lack of political know-how; NIMBYism from affluent classes	Affluent NIMBYism and lack of political know-how block bridging capital and secure land tenure for underserved groups.
Shackleton et al. (2024) (34)	South Africa	Slum dwellers, foragers	Leftover spaces; Unsanctioned occupation	High transport costs	Apartheid-era spatial legacies; Siloed governance	Apartheid-era legacies and siloed governance force slum dwellers into unsanctioned leftover spaces with high transport costs.
Shostak (2022) (38)	USA	Black and Brown farmers	Toxic legacies (heavy metals); Embodied disconnection	Cost-prohibitive soil remediation	Environmental racism; Redlining legacies	Institutional legacies of redlining and environmental racism result in toxic soil conditions that are cost-prohibitive to remediate.
Silva et al. (2018) (32)	Estonia / Portugal	Roma minorities, migrants	Abandoned industrial zones; Peripheral isolation	Rent increases; Eco-gentrification	Deep spatial segregation; Displacement	Deep social segregation and eco-gentrification drive rent increases, forcing minorities into isolated, abandoned industrial zones.
Threlfall et al. (2022) (50)	Australia	Renters, non-English speakers	Unequal tree distribution	Renting status barriers	Procedural inequity; Internal municipal bias	Procedural inequity and municipal biases against renters manifest structurally in unequal tree distribution and physical access barriers.
Truong et al. (2022) (36)	Australia	Social housing tenants	Cramped interstitial spaces	Individual financial precarity	Stigmatization of marginalized lifestyles	The stigmatization of marginalized lifestyles and individual financial precarity restrict tenants to cramped interstitial spaces.
Wardle et al. (2024) (1)	UK	Vulnerable therapy groups	Complex land use permissions	Continuous grant-seeking cycle	Volunteer burnout; Pandemic-induced isolation	Pandemic-induced isolation and volunteer burnout perpetuate a continuous, precarious grant-seeking cycle for land use permissions.
White and Bunn (2017) (43)	UK	Unemployed, activists	Lack of permanent tenure; Demolition threats	Patchwork funding cycles	Interim use designation; Bureaucratic exhaustion	Bureaucratic exhaustion and patchwork funding relegate activists to interim use spaces under constant threats of demolition.
Witheridge and Morris (2016) (18)	UK	Socially isolated groups	Rigid mono-zoning	Reliance on short-term grants; Mission creep	Neoliberal policies; Lack of collective rights	Neoliberal policies and lack of collective rights force a reliance on short-term grants within rigid, exclusionary mono-zoning frameworks.
Wolch et al. (2014) (9)	USA / China	Low-income, racial minorities	Proximity to environmental hazards	Public underfunding in deprived areas	Eco-gentrification; Green space paradox	Eco-gentrification and the green space paradox result in public underfunding and increased proximity to hazards for racial minorities.
Zhang et al. (2025) (6)	China	Older adults residents	Encroachment by built areas; Spatial fragmentation	Luxury effect	Legacy effects of planning; Pro-development bias	Institutional pro-development biases and the luxury effect drive spatial fragmentation and encroachment on older adults residents’ green spaces.
Zhang et al. (2021) (49)	China	Low-income communities	Extremely limited and scattered spaces	Housing displacement	Social segregation; Class-based exclusion	Class-based exclusion and economic housing displacement leave low-income communities with extremely limited, scattered spaces.

While Table 1 details these localized manifestations, mapping the intersectional flow between specific marginalized identities and their corresponding mechanisms of exclusion further elucidates the broader systemic dynamics. To visualize these complex entanglements, a Sankey diagram was developed (Figure 3). This mapping demonstrates how distinct socio-demographic groups converge upon shared structural hurdles through vastly different pathways of marginalization. By tracing these flows from vulnerable populations to overarching systems of power, the visual synthesis indicates that while low-income and working-class cohorts are predominantly funneled toward physical land tenure precarity and the threat of eco-gentrification, refugees and transnational migrants navigate a deeply entrenched network of institutional stigmatization and epistemic exclusion.

**Figure 3:**
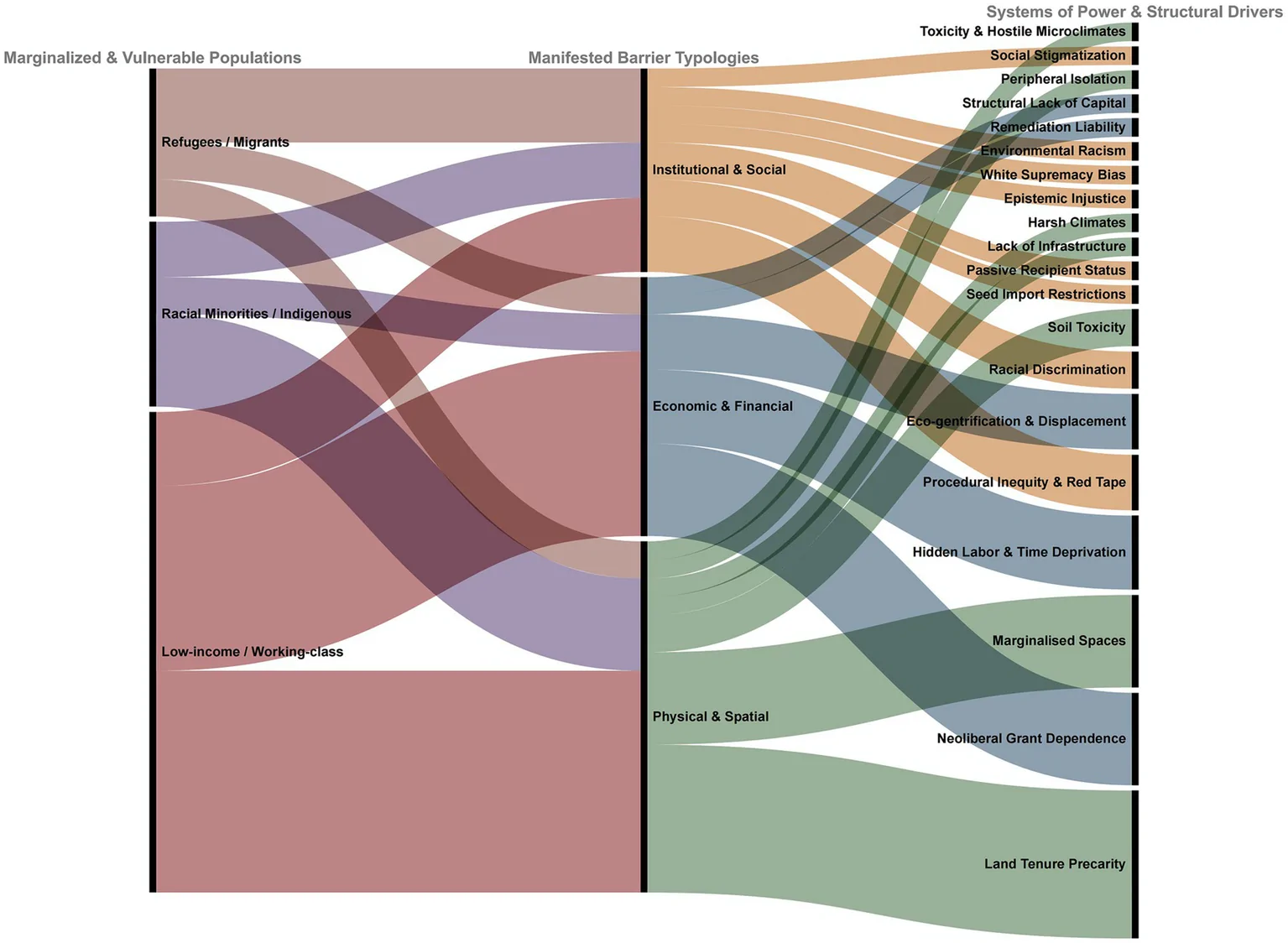
Sankey diagram illustrating the intersectional flow between vulnerable populations and the triad of structural barriers. This visualization exposes the multi-threaded entanglement between marginalized identities and granular mechanisms of spatial and procedural exclusion. Our analysis indicates that while low-income and working-class cohorts are predominantly affected by physical land tenure precarity and the threat of eco-gentrification, refugees and transnational migrants navigate a complex network of social stigmatization and systemic epistemic exclusion.

#### 3.2.1 Physical and spatial barriers: the precarity of healing grounds

The literature identifies the lack of secure physical space as the most fundamental barrier to therapeutic UA. The synthesized evidence demonstrates that this physical deficit reflects systemic spatial deprivation rather than a mere logistical hurdle (31, 42). The reviewed studies highlight that physical barriers impede the healing potential of these spaces through three intersecting mechanisms: land tenure precarity, relegation to fragmented geographies, and exposure to environmental toxicity (34, 38).

##### 3.2.1.1 The ephemerality of land tenure and the threat of displacement

The extracted data indicate that land tenure precarity severely destabilizes grassroots greening initiatives. With upwards of 80% of urban community gardeners cultivating land they do not own, the survival of these spaces remains entirely at the discretion of landowners (42, 43). As noted in the contexts of rapidly densifying cities, speculative reinvestment frequently consumes vacant plots, which the literature suggests directly exacerbates the green gentrification paradox for the urban poor (17, 44). Consequently, this spatial instability induces significant psychological stress. Evidence shows that the absence of long-term tenure guarantees actively deters marginalized groups from investing physical and emotional labor into spaces vulnerable to unannounced redevelopment (31, 35).

##### 3.2.1.2 Relegation to leftover and fragmented geographies

Driven out by competitive real-estate markets, therapeutic agriculture is frequently relegated to the spatial margins. This marginalization is conceptualized within the evidence base as territorial fragmentation. For example, in peri-urban Italy, rapid urbanization systematically reduces accessible agricultural land (13), while in Sydney’s social housing estates, green infrastructure is confined to cramped interstitial spaces adjacent to parking lots or back alleyways (36). The synthesis reveals that these spatial constraints become particularly acute in the Global South. Amidst the density of Kampala’s informal settlements and South Africa’s apartheid-era spatial legacies, informal housing rapidly consumes open land, leaving minimal physical capacity for therapeutic engagement (34, 45). By being relocated to peripheral or former industrial zones, minority and low-income residents face considerable geographical distances that fundamentally sever their physical access to nature (32, 46).

##### 3.2.1.3 Environmental toxicity and microclimatic hostility

However, the review demonstrates that merely securing land does not guarantee therapeutic efficacy, as the physical environment itself frequently presents severe challenges. In Boston, relict industrial sites situated near Black and Brown communities contain historically toxic soils, forcing marginalized farmers to import costly clean soil and construct raised beds (38). This mandatory remediation effectively severs the direct, embodied connection to the earth universally associated with horticultural recovery. Furthermore, adverse microclimatic conditions heavily degrade therapeutic value. Synthesizing evidence from unpredictable water restrictions in Native American reservations (37) to the short growing seasons confronting African immigrants in Canada (40), it becomes evident how hostile physical environments transform intended refuges into highly stressful transitional spaces, thereby undermining both agricultural viability and social cohesion (9, 47).

#### 3.2.2 Economic and financial barriers: the eco-gentrification paradox

Our synthesis establishes that economic constraints in therapeutic UA extend far beyond operational budget shortfalls; rather, they represent a profound structural exclusion driven by neoliberal austerity, the eco-gentrification paradox, and a systemic reliance on uncompensated labor.

##### 3.2.2.1 The neoliberal trap of competitive funding

Our analysis shows that municipal divestment and rigid fiscal policies chronically limit public funding for grassroots initiatives (44, 48). Consequently, garden organizers face a continuous cycle of grant-seeking, remaining heavily reliant on competitive, short-term external capital (1, 18). We identified that this precarious funding structure not only creates significant administrative burdens but also triggers mission creep, compelling grassroots projects to compromise their foundational therapeutic goals to align with the restrictive metrics of external funders (18, 43). Furthermore, the reviewed literature demonstrates that systemic inequality heavily skews the distribution of this capital. For instance, Black-led urban farms must navigate far greater structural hurdles to secure basic startup funding compared to their white-led counterparts, exposing deep-seated racial disparities within mainstream agricultural financing (17).

##### 3.2.2.2 The paradox of eco-gentrification and the luxury effect

A critical spatial paradox emerges within the evidence base when successful greening projects inadvertently disadvantage vulnerable communities by triggering eco-gentrification (9). The synthesis observes that the installation of high-quality green infrastructure routinely inflates neighborhood attractiveness and property values, ultimately displacing the exact low-income residents these gardens were originally designed to serve (32, 46, 49). The documented luxury effect further compounds this exclusion. Because affluent neighborhoods generally command greater municipal investment, high-income residents can freely cultivate ornamental landscapes for aesthetic and psychological benefits. In stark contrast, the literature notes that low-income gardeners, burdened by immediate economic survival pressures, are forced to prioritize crop cultivation for basic food security, often overriding the potential for therapeutic engagement (42, 50).

##### 3.2.2.3 The hidden exploitation of time and volunteer labor

The synthesized evidence reveals that the mainstream narrative celebrating UA’s low entry barriers routinely obscures the severe economic demands placed upon the urban poor. Sustaining a community garden requires immense physical and temporal labor—resources that economically marginalized populations critically lack. For example, African immigrants in Edmonton, managing multiple minimum-wage jobs amidst high living costs, find the unpaid temporal commitment for collective gardening nearly impossible to sustain (40). Similarly, within Kampala’s informal settlements, the marginal livelihood yields from micro-gardens frequently fail to justify the extensive labor required for their maintenance (45). Consequently, the overarching literature indicates that the sustainability of these therapeutic spaces is often built upon the hidden exploitation of unpaid volunteerism from vulnerable groups, rather than the provision of secure, compensated horticultural management positions (47, 51).

#### 3.2.3 Institutional and social exclusion: invisible fences

Beyond tangible deficits in land and capital, the synthesized evidence identifies a complex web of institutional and social barriers operating as invisible fences against marginalized communities. The literature links the exclusion from therapeutic UA directly to procedural inequity, social stigmatization, and the cynical integration of green spaces into broader neoliberal economic agendas.

##### 3.2.3.1 Procedural inequity and bureaucratic disconnection

The synthesis establishes procedural exclusion as a primary institutional barrier. Path dependency and bureaucratic silos routinely hinder urban green space governance, severely limiting cross-sectoral coordination (13, 44). Extracted data reveal that this structural rigidity disproportionately penalizes vulnerable populations. In Melbourne, planners systematically omit residents with limited English proficiency from green infrastructure consultations, effectively stripping them of spatial agency (50). Simultaneously, grassroots leaders often lack the specialized institutional knowledge required to navigate labyrinthine zoning codes (18). Furthermore, the analysis reveals how affluent cohorts leverage procedural vulnerabilities to enforce exclusion. In Tampa, upper-income residents utilized their political capital to veto community gardens in adjacent low-income neighborhoods, employing neighborhood aesthetics and property values as justifications to maintain strict spatial boundaries (35).

##### 3.2.3.2 Social stigmatization and environmental inequities

Institutional exclusion is also directly tied to entrenched racial and class-based stigmatization. The literature demonstrates that mainstream urban greenery is frequently coded as an exclusionary landscape, subjecting migrants and ethnic minorities to overt social hostility and discrimination (39, 40). Building upon Forbes’ (17) critique, the evidence suggests that when mainstream agricultural initiatives adopt a stance of political neutrality, they actively obscure the historical disinvestment inflicted upon minority communities. Documented cases illustrate that this stigmatization physically and psychologically alienates marginalized groups. In Accra, local authorities consistently devalue indigenous farmers by classifying them as temporary squatters, thereby legally undermining their spatial rights (52). Similarly, social housing tenants in Sydney must battle external stereotypes—being labeled as socially deviant or mentally unstable—which compound their isolation within the very spaces designed for their healing (36).

##### 3.2.3.3 Institutional co-optation and the interim use designation

Finally, the reviewed studies highlight the institutional co-optation inherent in the precarious designation of community gardens within urban planning frameworks. Municipal governments frequently classify therapeutic agriculture as interim uses—temporary stopgap measures permitted strictly until the land becomes viable for lucrative commercial development (42, 43). The synthesis exposes how top-down municipal greening interventions are frequently deployed as mechanisms for urban revitalization that prioritize standardized aesthetics over informal, community-led land uses. Ultimately, these state-sanctioned interventions artificially inflate property values and accelerate the physical displacement of the low-income residents they purportedly serve (41).

## 4 Discussion

### 4.1 Theoretical reflections: beyond the green myth and towards healing justice

Our findings critically challenge the depoliticized green myth—the assumption that the mere physical provision of urban agriculture (UA) inherently guarantees equitable therapeutic outcomes—a narrative that frequently ignores the complex socio-economic drivers shaping green space distribution (6). While recent systematic reviews robustly validate the clinical efficacy of horticultural therapy in alleviating depression and anxiety (3, 22), and extensive bibliometric analyses underscore the vital link between urban gardening and public health (21), we argue that this physiological focus remains structurally incomplete. We observe that when therapeutic landscapes are constrained by transient land tenure, competitive funding cycles, and systemic inequalities, they cease to function as accessible sanctuaries and instead become contested spaces that exacerbate spatial deprivation. For instance, building upon evidence of indigenous practitioners categorized as squatters in Accra and minority groups displaced by green gentrification in Europe, we find that the physiological benefits of horticultural activity are frequently offset by the precarity of physical eviction and spatial marginalization (32, 52).

The identification of this structural triad aligns with and significantly expands upon recent systematic reviews in allied disciplines. While prior reviews on urban green spaces have documented macro-level eco-gentrification and environmental racism (15, 16), and systematic reviews on nature-based solutions emphasize overarching systemic exclusions (24), our synthesis explicitly bridges these macro-dynamics with the localized, embodied practices of therapeutic agriculture. By connecting these previously isolated bodies of literature, we demonstrate that clinical benefits cannot be fully realized without simultaneously dismantling these intersecting structural barriers.

Addressing the intersectional pathways of marginalization mapped in our synthesis (Figure 3), we contend that transitioning from localized interventions to sustainable public health infrastructure necessitates a structural framework of healing justice (53). In marginalized contexts, we conceptualize UA not merely as the passive consumption of green amenities, but as a critical spatial practice and a form of rooted resistance against systemic marginalization and eco-gentrification. For community-led urban farms navigating systemic divestment, land cultivation represents more than horticultural engagement. We identify these spaces as vital compass institutions, enabling communities to reclaim spatial agency over historically disinvested or remediated lands (17, 38). Ultimately, we conclude that therapeutic outcomes for vulnerable populations cannot be evaluated solely through physiological metrics. True efficacy depends on dismantling the structural invisible fences that dictate access to and governance of the healing city. To conceptualize this transition, we propose a multidimensional framework for healing justice (Table 2).

**Table 2:** A multidimensional framework for healing justice: operationalizing systemic interventions against structural exclusion in UA.

Targeted barrier	Paradigm shift	Specific policy and practice interventions
1. *Physical and spatial precarity*: land tenure insecurity and leftover geographies	Spatial de-commodification and permanent tenure	*Community land trusts (CLTs)*: establishing non-profit community trusts to secure permanent land stewardship, explicitly protecting marginalized growers from speculative displacement (38). *Just green enough strategies*: enacting anti-displacement zoning that prioritizes small-scale, community-led interventions over large-scale ecological redevelopments that catalyze eco-gentrification (9, 46). *Infrastructure Re-categorization*: legally re-coding therapeutic UA as essential civic infrastructure to transition away from precarious interim use designations (43).
2. *Economic and financial strangulation*: neoliberal austerity and labor exploitation	Structural de-marginalization and capital redistribution	*Core municipal funding*: reducing the reliance on highly competitive, short-term grants by integrating UA into baseline public health and urban planning budgets (13). *Compensated labor models*: instituting fair wages and stipends for grassroots organizers to address the reliance on unpaid temporal labor from vulnerable populations (40, 47). *Unrestricted grant pools*: establishing revolving, unrestricted financial capital—particularly for minority-led initiatives—to address disparities inherent in traditional competitive funding metrics (17).
3. *Institutional and epistemic violence*: procedural exclusion and socio-cultural marginalization	Co-governance and epistemic justice	*Inclusive Food Policy Councils (FPCs)*: Mandating that historically displaced populations hold formal, binding decision-making authority in urban resource allocation (51). *Epistemic validation and seed sovereignty*: formally integrating indigenous agricultural practices into municipal frameworks and reconsidering restrictive seed policies that affect transnational migrants (37, 40). *Trauma-informed spatial design*: cultivating physically and culturally safe sanctuaries that mitigate surveillance and socio-spatial exclusion (35, 41).

This framework conceptualizes a structural transition from managing physiological symptoms to addressing root structural causes. We argue that by operationalizing these interventions, urban policy must shift from demanding individual resilience to facilitating structural healing justice through equitable spatial governance.

### 4.2 Policy and practice implications: dismantling the three barriers

We argue that transitioning UA from a precarious interim use into a permanent therapeutic infrastructure requires a decisive shift away from fragmented, short-term interventions. As operationalized in our proposed tripartite framework (Table 2), addressing these structural deficits necessitates targeted policy responses across physical, economic, and institutional domains.

#### 4.2.1 Securing spatial agency: community land trusts and just green enough strategies

We posit that addressing spatial deprivation necessitates comprehensive legal frameworks that protect therapeutic spaces from speculative real estate markets. Establishing Community Land Trusts (CLTs), municipal land banks, and public peri-urban agricultural parks (13) can systematically transfer spatial agency to marginalized growers, converting precarious plots into legally protected commons for sustainable planning. For historically marginalized farmers navigating remediated industrial sites, such as those in Boston, we highlight how CLTs provide the requisite tenure security to invest in soil remediation without the immediate risk of displacement (38). Furthermore, to mitigate the eco-gentrification paradox, we strongly recommend that municipalities adopt “just green enough” anti-displacement zoning strategies. This approach explicitly prioritizes small-scale, community-led interventions tethered to rent stabilization policies, thereby ensuring that working-class neighborhoods are not displaced by the landscape improvements intended to support them (9, 32, 46).

#### 4.2.2 De-commodifying healing: long-term funding and compensated stewardship

Our synthesis underscores that the financial sustainability of therapeutic spaces requires a decisive shift toward stable, long-term funding models. Treating community gardens as core civic health infrastructure can drastically reduce reliance on short-term competitive grants—a dependency that we observe frequently leads grassroots organizations, such as those in Edinburgh, into administrative exhaustion and unintended mission creep to satisfy restrictive funder requirements (1, 18). Furthermore, we argue that the systemic reliance on uncompensated labor from marginalized groups must be dismantled. Sustaining these spaces requires instituting compensated horticultural management positions, rather than extracting unpaid volunteerism from populations already facing extreme economic precarity (40, 47). For minority-led farms historically impacted by systemic disinvestment, we suggest that capital redistribution can be facilitated through unrestricted grant pools and participatory budgeting. This targeted approach bypasses traditional, highly competitive funding metrics, effectively ensuring more equitable access to therapeutic resources (17, 48).

#### 4.2.3 Epistemic justice and co-governance: dismantling institutional fences

Our analysis indicates that overcoming institutional exclusion involves directly addressing procedural inequity and top-down bureaucratic structures by pivoting toward inclusive mosaic governance (24). We recommend mandating broadly representative Food Policy Councils (FPCs) to embed procedural justice within municipal decision-making, ensuring that historically displaced populations possess formal, binding authority over local land-use agendas (43, 51). Moreover, we emphasize that genuine systemic healing requires epistemic justice, which involves formally validating indigenous and localized knowledge systems. By elevating the decision-making power of Indigenous tribal leaders in agricultural policies in Montana (37), or by formally recognizing indigenous growers in Accra as legitimate spatial actors rather than criminalized squatters (52), policy frameworks can successfully challenge exclusionary knowledge hierarchies. We conclude that by empowering minority-led urban farms as recognized community institutions, cities can transform marginalized spaces into resilient environments of collective spatial agency (17, 36).

### 4.3 Limitations of the scoping review

Methodological boundaries inherently shape the scope of our review. In accordance with JBI and PRISMA-ScR frameworks (25, 26), we prioritized mapping the breadth of structural and procedural barriers over synthesizing individualized clinical effect sizes; thus, we omitted formal risk-of-bias appraisals. Furthermore, we acknowledge that restricting our search to English-language peer-reviewed articles indexed in Web of Science and Scopus introduces a recognized geographic and language bias. This approach tends to privilege the socio-spatial dynamics of the Global North, disproportionately representing structural challenges such as industrial legacies in Boston or grant dependencies in Edinburgh (18, 38). Consequently, our synthesis may underrepresent localized empirical evidence and grassroots policy reports from the Global South. We also recognize that omitting gray literature risks overlooking the informal spatial tactics deployed in Francophone African contexts or Latin American settlements. Beyond language and database limitations, an inherent epistemic bias stems from our search terminology itself. The concept of “therapeutic urban agriculture” is predominantly mobilized by urban planning frameworks within the Global North. This Eurocentric framing can act as an epistemic euphemism that inadvertently obscures alternative socio-political realities. For instance, in many parts of Latin America, comparable initiatives are frequently conceptualized not as “therapeutic” interventions, but as “community vegetable gardens” (e.g., huertas comunitarias). As critical scholarship highlights, these gardens often emerge from the structural precariousness of urban peripheries, operating as essential survival strategies and spatial acts of social and political resistance (54). Consequently, our reliance on the term “therapeutic” largely explains the stark underrepresentation of Latin American empirical studies, emphasizing the urgent need for future global reviews to decolonize search typologies and incorporate context-specific terminologies. Ultimately, the structural barriers and overarching claims synthesized in this review must be explicitly bounded to the Global North evidence base, and extreme caution should be exercised before extrapolating these frameworks globally.

### 4.4 Future trends and research directions

Expanding research beyond the Global North remains an empirical necessity. We encourage future inquiries to deeply investigate informal settlements in the Global South, where spatial deprivation and climate vulnerability frequently intersect. We suggest that tracking how youth in Kampala’s K-zones mobilize micro-agriculture to reclaim marginalized spaces offers a necessary counter-narrative to Eurocentric models of eco-gentrification (34, 45).

Methodologically, we identify a critical need to transition from cross-sectional snapshots to longitudinal studies. We argue that evaluating structural interventions requires tracking marginalized populations across extended temporal horizons to assess, for example, how securing permanent land tenure through CLTs mitigates the long-term impacts of industrial legacies for Black urban farmers, or how consistent municipal funding breaks the cycle of administrative burden (18, 38).

Finally, we recommend that future spatial analyses adopt a robust intersectional approach to decode the compounded weight of identity and geography. Future scholarship must examine how intersecting vulnerabilities—such as refugee status, race, and economic precarity—magnify institutional barriers. Investigating how transnational migrants navigate restrictive seed policies alongside significant economic constraints will provide a clearer, highly localized understanding of the procedural exclusions embedded in contemporary environmental planning (32, 40).

## 5 Conclusion

Our scoping review critically dismantles the depoliticized green myth surrounding therapeutic UA. By synthesizing 30 core empirical studies, we demonstrate that the healing potential of green infrastructure is fundamentally constrained by a triad of structural barriers. The synthesized evidence establishes that for vulnerable populations, the therapeutic benefits of cultivating land are routinely undermined by systemic obstacles. Specifically, marginalized growers must navigate spatial precarity within fragmented or remediated geographies (13, 38), economic constraints tied to competitive grant cycles and administrative burdens (18), and procedural exclusion stemming from systemic inequities and institutional stigmatization (17, 36).

We conclude that the efficacy of horticultural interventions remains fundamentally limited when situated within contexts of spatial injustice, particularly within the Global North contexts from which our evidence is predominantly drawn. Our synthesis indicates that implementing localized greening projects without addressing broader structural vulnerabilities is often counterproductive, particularly as marginalized populations face concurrent risks of speculative eco-gentrification and procedural marginalization. To transition UA from an exclusionary amenity to an inclusive public health asset, we argue that comprehensive structural intervention is required. We urge urban policymakers to prioritize the institutionalization of Community Land Trusts, establish stable core funding, and implement co-governance models to secure spatial agency for marginalized communities. Furthermore, fostering spatial justice necessitates moving beyond top-down frameworks to embrace community-led approaches that ensure equitable climate resilience and local ownership of neighborhood change (55). Ultimately, by dismantling these invisible fences and integrating a healing justice framework into the foundations of urban planning, cities can cultivate inclusive environments that support collective resilience, spatial equity, and systemic well-being.

## Data Availability

The original contributions presented in the study are included in the article/Supplementary material, further inquiries can be directed to the corresponding author.
