# Supplementary material for: Beyond the green myth: a scoping review of spatial justice and structural barriers to therapeutic urban agriculture for vulnerable communities
Source: Front Public Health. 2026 Sep 29;14:1907463. doi: 10.3389/fpubh.2026.1907463 (PMC13658620; doi:10.3389/fpubh.2026.1907463)
Supplement: Supplementary file 1 [file Table_1.docx]

**Supplementary Material**

**Appendix A**

**Methodological Transparency Report**

**A.1. Full Database Search Strategies**

Table A1 details the search syntax, applied parameters, and record yields for our systematic retrieval process. To prevent indexing discrepancies, we executed all database searches concurrently on March 18, 2026.

**Table A1.** Detailed Search Strategy and Retrieval Outcomes.

| **Database** | **Execution Date** | **Primary Search Syntax** | **Applied Parameters** | **Yield (n)** |
| --- | --- | --- | --- | --- |
| Web of Science  (Core Collection) | March 18, 2026 | TS= ("urban agriculture" OR "horticultural therapy" OR "community garden" OR "urban farm*" OR "rooftop garden*" OR "therapeutic garden*") AND TS= ("spatial equity" OR "spatial access*" OR "environmental justice" OR "policy" OR "zoning" OR "implementation barrier*" OR "governance" OR "land use" OR "spatial planning" OR "exclusion") AND TS= ("mental health" OR "wellbeing" OR "well-being" OR "psychological") | Time Span: All years (Inception to March 18, 2026)  Document Type: Article OR Review  Language: English | 90 |
| Scopus | March 18, 2026 | TITLE-ABS-KEY (("urban agriculture" OR "horticultural therapy" OR "community garden" OR "urban farm*" OR "rooftop garden*" OR "therapeutic garden*") AND ("spatial equity" OR "spatial access*" OR "environmental justice" OR "policy" OR "zoning" OR "implementation barrier*" OR "governance" OR "land use" OR "spatial planning" OR "exclusion") AND ("mental health" OR "wellbeing" OR "well-being" OR "psychological")) | Time Span: All years (Inception to March 18, 2026)  Document Type: Article OR Review  Language: English | 214 |
| Total Records Retrieved |  |  |  | 304 |

**A.2. PRISMA-ScR Compliance Statement**

We conducted this scoping review in accordance with the Preferred Reporting Items for Systematic reviews and Meta-Analyses extension for Scoping Reviews (PRISMA-ScR) guidelines [25]. We maintain a completed 22-item PRISMA-ScR checklist as a supplementary file, which cross-references all mandatory reporting standards with their corresponding manuscript sections.

*Note:* The PRISMA Flow Diagram (Figure 1, main manuscript) illustrates the screening protocol for the initial 304 records, deduplication procedures, and full-text exclusion rationales.

**Appendix B**

**Comprehensive Evidence Matrix of Included Studies (n=30)**

Data from the 30 included studies were systematically charted to map targeted populations, structural barriers, and proposed interventions. These structural barriers are categorized into Physical and Spatial Barriers, Economic and Financial Barriers, and Institutional and Social Exclusion, aligning directly with the thematic framework of this scoping review.

**Table B1.** Systematic Data Extraction of Structural Barriers and Proposed Solutions in Therapeutic Urban Agriculture.

| **Study** | **Location** | **Population** | **Methodology** | **Type of UA** | **Physical and Spatial Barriers**  **(Evidence)** | **Economic and Financial Barriers (Evidence)** | **Institutional and Social Exclusion (Evidence)** | **Proposed Solutions** | **Core Finding and Critical Quote** |
| --- | --- | --- | --- | --- | --- | --- | --- | --- | --- |
| Allen and Apsan Frediani (2013) [52] | Accra, Ghana | Urban poor, indigenous farmers | Case study; action learning | UPA, vegetable farming | Encroachment of Customary Land: Displacement to remote peripheries.  (“large tracks of customary land enter into the real-estate market... displacement of farming to further away areas”) | Corporate Dependency Cycles: Forced reliance on artificial inputs and hybrid seeds.  (“trap farmers in a 'cycle of dependency' in which they need to purchase inputs”) | Stigmatization as Squatters: Devaluation of indigenous knowledge.  (“labelled by local developers as squatters... reproducing the hierarchy and supremacy of technical knowledge”) | Designation of UA as passive land; Utilization of UA as a spatial political practice for the right to the city. | **Core Finding:** UA is stigmatized as illegal occupation, trapping farmers between land grabs and corporate dependency.  *"Rather than approaching UA as an alternative livelihood... we approximated it as a spatial practice... to disrupt existing relations of dependency."* |
| Bailey and Kingsley (2022) [31] | Melbourne, Australia | Local residents, migrants | Qualitative interviews; political economy analysis | Community / allotment gardens | Land Tenure Precarity: Obstacles in securing land amid densification.  (“A significant obstacle to urban gardening is first gaining access to land and subsequently securing ongoing access”) | Neoliberal Sell-offs and Underfunding: Commercialization of public green spaces.  (“Securing land... within a neoliberal context of selling off community gardens... issues pertaining to funding”) | Pro-Sports Municipal Bias: Marginalization of UA resource allocation.  (“it's currently disproportionate, the amount of resources that go into those sporting areas”) | Implementation of grassroots commoning practices; Scaling up UA into formal institutional structures. | **Core Finding:** Neoliberal densification and pro-sports bias subject community gardens to constant speculative threats.  *"Securing land for urban gardening within a neoliberal context of selling off community gardens... can prevent many who wish to create urban gardens from doing so."* |
| Brown et al. (2020) [37] | Montana, USA | Native Americans (AI/NA) | CBPR; social-ecological model | Community gardens | Hostile Microclimates and Water Scarcity: Rocky soils and tribal water restrictions.  (“Soil was imported... high in rock and clay... poor access to water was cited as a major barrier”) | The Food Subsidy Trap: Poverty-driven reliance on cheap processed foods.  (“Food prices are already cheap so it’s not worth it... convenience of using food stamps over growing food”) | Generational Disconnect: Federal food policies suppressing traditional agriculture.  (“existing federal and state policies make it convenient for tribal members to buy cheap processed foods”) | Rebuilding trust via CBPR; Intergenerational knowledge transfer via tribal Elders; Enactment of agricultural water policies. | **Core Finding:** Traditional UA revival on reservations is stifled by a policy-driven addiction to subsidized processed foods.  *"Existing federal and state policies make it convenient for tribal members to buy cheap processed foods and thus forgo buying/growing local foods."* |
| Contesse et al. (2018) [44] | Santiago, Chile | Low-income residents | Policy Arrangement Approach (PAA) | Urban Agriculture | Siting Marginalization: Relegation to disused, unprofitable urban spaces.  (“limited to disused urban spaces without a more profitable use”) | Municipal Budget Constraints: Unaffordable maintenance costs for local governments.  (“UA is more expensive and laborious to maintain... finding municipalities that count with the budget”) | Public-Private Conflicts: Stigmatization as private space appropriation.  (“unclear to what extent UA sites can function as public spaces... appropriation of the public space”) | Formal integration into green space definitions; Implementation of land-leasing frameworks for collective management. | **Core Finding:** UA is stigmatized as an expensive private burden within fragmented urban governance.  *"It is unresolved who is involved and who benefits from urban agriculture."* |
| Egoz and De Nardi (2017) [39] | Global / Europe | Refugees, Migrants | Literature review | Urban Parks and Gardens | Practical Transit Obstacles: Distant green spaces and lack of multilingual signage.  (“experience practical obstacles such as lack of multilingual signs, or parking problems”) | Socio-economic Marginality: Income-based deprivation limiting access.  (“Marginality refers to socio-economic barriers... such as low incomes”) | White Landscape Ideals: Racial discrimination and exclusionary spatial constructs.  (“exclusion of minorities from an idealised landscape considered essentially 'white'”) | Adoption of landscape justice frameworks; Engagement of landscape experts in refugee resettlement planning. | **Core Finding:** Migrants face a triple burden of spatial, economic, and racial exclusion from idealized white landscapes.  *"Social sustainability is viable only within an ethos of equality and justice... landscape is a common good."* |
| Fanfani et al. (2024) [13] | Prato, Italy | Local residents | Territorialist approach; Break-even analysis | Peri-urban Agricultural Park | Territorial Fragmentation: Loss of accessible agricultural land to urbanization.  (“fragmentation of the territory... poor accessibility of green areas”) | Financial Inviability: Absolute dependence on public subsidies for survival.  (“premises for requiring the support of a public contribution”) | Bureaucratic Silos: Administrative path dependence hindering innovation.  (“aligned on path dependence models and articulated by 'silos'”) | Establishment of negotiated Public-Private Partnerships (PPPs); Integration of income-generating cross-subsidies. | **Core Finding:** Rigid bureaucratic silos and financial deficits stifle peri-urban park implementation.  *"The project poses a non-negligible challenge for the action of public administration... articulated by 'silos'."* |
| Ferreira et al. (2020) [24] | Global | Low-income groups | Systematic Literature Review | Nature-based Solutions (NBS) | Complex Spatial Deficits: Severe lack of skilled planning personnel in dense cities.  (“Lack of skilled personnel/technical and scientific knowledge”) | Financial Constraints and Time Poverty: Inability of low-income citizens to afford participation time.  (“citizens with low income may find it difficult to devote time to this type of process”) | Bureaucratic Red Tape: Institutional hierarchies and low social cohesion.  (“Understand the hierarchies of institutions... Deal with conflicting points of view... Overcome the poor flow of information”) | Creation of cross-sectoral learning alliances; Application of mosaic governance to enhance marginalized participation. | **Core Finding:** Conflicting interests and financial constraints systematically exclude marginalized groups from NBS implementation.  *"Expectations regarding the implementation of NBS are detached from the real challenges faced through their implementation."* |
| Forbes (2024) [17] | USA | Black urban farmers | Comparative case study | Black-led urban farms | Speculative Dispossession: Loss of vacant land due to reinvestment in historically Black neighborhoods.  (“urban divestment, speculative reinvestment, and displacement-based gentrification... reduced the availability of vacant land”) | Racial Capitalism Barriers: Structural funding gaps aimed at elite value extraction.  (“racial capitalism... devalue Black and Brown lives and spaces... struggle to access financial capital”) | Apolitical White-Dominance: Mainstream UA masking systemic racial violence.  (“public discourse... shaped by white actors... publicly apolitical initiatives mask the white supremacy”) | Recognition of Black-led farms as compass institutions; Provision of unrestricted grant funding for anti-racist placemaking. | **Core Finding:** Black-led farms serve as compass institutions navigating ruins left by racial capitalism.  *"Black-led urban farms function as compass institutions, charting paths to Black community liberation... from ingrained white supremacy."* |
| Furlong et al. (2025) [46] | Nottingham, UK | Working-class, low-income | Ethnography; IPA | Allotment gardening | Spatial Deprivation: High travel distances and physical isolation via high hedges.  (“having to travel a greater distance to access nature... isolated on rows away from the main path”) | Eco-gentrification Threats: Rising living costs contrasting with accessible allotments.  (“increases in urban greenery... has caused increases in cost of living... it’s cheaper than therapy”) | Gang-related Surveillance: Social anxiety and perception of green spaces as unsafe.  (“viewed as being low quality and unsafe, due to the prevalence of gang-related activity”) | Implementation of just green enough strategies; Empowerment of local control via communities of practice. | **Core Finding:** For the working class, allotments act as a vital dual refuge from mental trauma and eco-gentrification.  *"We can’t afford to go private... it’s cheaper than therapy and it’s cheaper than the studio space."* |
| Kenner et al. (2022) [41] | Philadelphia, USA | Black and Brown communities | Ethnography | Guerrilla gardens and Vacant lots | Physical Fencing Constraints: Fences used to conceal informal community land uses.  (“Fences conceal the land inside... vacant urban land operates as a problem-space”) | Maintenance Liabilities: Unaffordable repair costs transferred to low-income homeowners.  (“property maintenance... issues that often exceed the budgets of low-income homeowners”) | Performative Greening: Standardized cleaning deployed as disciplinary surveillance.  (“Greening... discourages and prevents land use coded unproductive... encouraged high-end real estate dev”) | Advancement of a politics of sight; Reframing of vacant lands as transformative community spaces. | **Core Finding:** Standardized municipal greening erases informal community uses to pave the way for capital accumulation.  *"Greening not only erases remnants of former ownership but also discourages and prevents land use coded unproductive."* |
| Kronsted (2024) [45] | Kampala, Uganda | Slum youth | Visual essay | Micro-urban agriculture | Ad-hoc Hyper-density: Extreme lack of open space and severe flood risks.  (“grown ad-hoc, leaving limited open space... The density leaves little room for trees”) | Livelihood Imbalances: Disproportionate labor-to-yield ratios in crowded slums.  (“the reward in terms of livelihood... did not compensate for the labour required”) | Systemic Deprivation: Political marginalization of youth in formal city planning.  (“spaces which have been deprived of social, spatial, and political privileges”) | Deployment of low-tech micro-gardening coupled with social skill training. | **Core Finding:** In dense slums, micro-agriculture acts as a spatial tactic for youth to reclaim public space despite minimal economic compensation.  *"The movement is using collective power to transform spaces... into sites of green, sustainable livelihoods."* |
| Langerman et al. (2025) [10] | Global | Refugees, immigrants | Scoping Review | Community and rooftop gardens | Material Resource Deficits: Severe lack of physical infrastructure and secure land access.  (“met with barriers such as access to land, infrastructure... lack the material resources”) | Unequal Funding Scales: Disparities between global North self-funding and global South aid reliance.  (“Differences in scale often relate to different funding sources”) | Procedural Exclusion: Passive recipient status lacking structural food justice frameworks.  (“health and food and environmental justice were applications that largely did not emerge”) | Integration of UA into broader urban systems; Examination of structural racism through a food justice lens. | **Core Finding:** Refugee interventions frequently fail due to top-down delivery models that ignore procedural inclusion.  *"There is a difference between an intervention for refugees... and an intervention with refugees... Future research could examine power dynamics."* |
| Li and Huo (2025) [33] | Shenzhen, China | Community residents | Case study; participatory design | Co-built Gardens | Complex Site Conditions: High spatial selection costs and commuting times in new urban districts.  (“High commuting and time costs further hinder collaboration with experienced design teams”) | Long Investment Cycles: Severe shortages in financial resources due to low economic returns.  (“long investment cycles and low economic returns... make community garden construction... complex”) | Fragmented Governance: Difficulty coordinating stakeholder interests over sustained maintenance.  (“typically emphasize the initial design and construction phases rather than sustained care”) | Establishment of university-driven collaborative governance to integrate design education with community service. | **Core Finding:** A university-centered model effectively bridges resource gaps in new districts via social practice.  *"The governance structure model... stands out from other approaches by positioning the university at the core of the process."* |
| Lu et al. (2025) [48] | China | Gated community residents, tenants | Mixed-methods | Gated Community Gardens | Legal Gray Areas: Lack of formal institutional support on collectively owned land.  (“developed on collectively owned land and lack formal institutional support; this presents a legal gray area”) | High Infrastructure Costs: Reliance on grassroots fundraising due to absent core budgets.  (“infrastructure... can be very expensive... residents turned to grassroots fundraising”) | Consensus Suppression: Over-emphasis on group harmony marginalizing dissenting voices.  (“strong emphasis on consensus and group harmony... can sometimes suppress dissenting voices”) | Strategic leveraging of collectivist discourse for political legitimacy; Dedicated participatory budgeting. | **Core Finding:** Grassroots gardens survive in legal gray areas by strategically attaching to collectivist narratives.  *"For grassroots efforts, legitimacy involves both political consent and public acceptance."* |
| Marsh et al. (2020) [47] | Hobart, Australia | University students, public | Mixed-methods | Campus Community Gardens | Hostile Microclimates: Cold, windy concrete environments blurring public-private boundaries.  (“cool microclimates... cold concrete blocks and few wind breaks”) | Transience and Underfunding: Overreliance on unpaid transient student volunteer labor.  (“overreliance on volunteerism from a transient student community... limited financial resourcing”) | Institutional Arrogance: Low community trust and severe university-community social friction.  (“university is perceived as irresponsible... display of arrogance and disrespect for community values”) | Execution of in-depth trust-building assessments; Creation of compensated facilitator roles. | **Core Finding:** Campus gardens risk becoming expensive ornaments without addressing hostile microclimates and institutional trust.  *"Without this understanding, potential significant barriers and enablers will go unnoticed."* |
| Otoadese et al. (2025) [40] | Edmonton, Canada | Black African Immigrants | CBPR | Collective Community Gardens | Severe Climate Barriers: Short growing seasons and lack of reliable transportation to sites.  (“cold weather and short growing season... lack of reliable means of transportation”) | Economic Survival Pressures: Time poverty induced by working multiple minimum-wage jobs.  (“working multiple minimum-wage jobs... limited time to engage”) | Systemic Racism: Restrictive seed import laws and culturally exclusionary interpersonal dynamics.  (“Systemic racism... Import restrictions on seeds from participants’ countries of origin”) | Investment in community greenhouse infrastructure; Reform of exclusionary seed importation policies. | **Core Finding:** Harsh climates intersect with systemic racism and labor exploitation to exclude African immigrants.  *"Experiences of discrimination... inadvertently reproduce broader social and structural patterns of inequity."* |
| Philpott et al. (2023) [42] | California, USA | Urban gardeners | Vegetation surveys, GLMs | Urban community gardens | Tenure Fragility: Widespread lack of land ownership limiting plot sizes and species richness.  (“upwards of 80% of urban community gardeners do not own the land that they cultivate”) | The Luxury Effect: Economic survival coercing low-income growers to prioritize food crops over ornamental aesthetics.  (“higher household income was associated with... higher flower abundance... low-income gardeners focus on growing crops”) | Disinvestment Cycles: Temporariness hindering long-term investments in soil health.  (“insecure land tenure may affect garden management by resulting in a sense of 'temporariness'”) | Legislation of permanent land tenure (public ownership/CLTs) to protect vulnerable food security. | **Core Finding:** Tenure security and the luxury effect dictate whether a garden produces ornamental landscapes or essential food.  *"Insecure land tenure may affect garden management by resulting in a sense of 'temporariness'... cultivated plants are strongly shaped by socioeconomic factors."* |
| Rao et al. (2022) [51] | Global | Urban poor, racial minorities | Systematic Literature Review | UPA, community gardens | Tenure Insecurity: Constant threats of expropriation and intense urban development pressures.  (“sold in anticipation of expropriation... under constant pressure of urban development”) | Exploitation of Volunteer Labor: Reliance on unpaid labor; lack of stable, compensated horticultural employment.  (“jobs were reported to be low-wage and temporary... use of volunteer labour rather than paid workers”) | Devaluation as Unreal Agriculture: Procedural exclusion of racial minorities by municipal planners.  (“disadvantaged groups and racial minorities are often excluded... UPA is then not seen as 'real agriculture'”) | Establishment of robust legal frameworks for agricultural land protection; Creation of inclusive Food Policy Councils. | **Core Finding:** Without tenure security, UPA benefits are captured by elites, systematically marginalizing the urban poor.  *"UPA is then not seen as 'real agriculture' by many stakeholders... who see it as a social rather than productive activity, hindering municipal planning."* |
| Raskin et al. (2021) [35] | Florida, USA | Historically underserved | Interviews, focus groups | Community / collective gardens | Tenure Insecurity: Political lip-service denying long-term land guarantees against future development.  (“lack of commitment from the political leaders to ensure tenure”) | Social Capital Deficits: Precarious reliance on bridging capital to access external donations.  (“bridging capital increased their access to local plant distributors... to provide us with soil”) | Elite NIMBYism: Upper-income sabotage of garden ordinances using discriminatory rhetoric.  (“upper income people who did not want somebody to start a community garden... wind up with piles of garbage”) | Facilitation training for community leaders in political know-how to navigate complex bureaucratic codes. | **Core Finding:** Low-income gardens face intense class-based sabotage and NIMBYism from affluent neighbors.  *"Those who actively campaigned against the development of the community garden ordinance were residents from the upper-income areas... they did not want a community garden in the lot next to their house."* |
| Shackleton et al. (2024) [34] | South Africa | Slum dwellers, foragers | Policy Impact Pathways | Urban agriculture, verges | Leftover Space Vulnerabilities: Rapid unsanctioned occupation of open land by backyard shacks.  (“available land tends to be viewed as 'left over' space... quickly covered with backyard shacks”) | Transport and Equipment Poverty: Financial inability to access distant public parks or purchase equipment.  (“poor households limited financial means constrains opportunities to travel to public parks”) | Apartheid-era Spatial Legacies: Siloed governance and procedural exclusion from formal design.  (“apartheid-era spatial planning intentionally entrenched patterns of dispossession... siloed governance”) | Adoption of pro-poor designs; Issuance of foraging permits; Integration of UA in spatial planning. | **Core Finding:** Apartheid spatial legacies and siloed governance subject the urban poor to severe procedural exclusion.  *"Yet, by virtue of being poor, they are frequently recognitionally or procedurally excluded from processes related to UGI design."* |
| Shostak (2022) [38] | Boston, USA | Black and Brown farmers | Ethnography | Urban Farms and Gardens | Toxic Legacies: Heavy metal-contaminated soils severely disconnecting farmers from the earth.  (“contaminated urban soils are disproportionately found in 'communities of color'”) | Liability Transfers: Government shifting of cost-prohibitive soil remediation burdens onto poor farmers.  (“shifting the cost of decades-long neglect back onto local farmers... significant financial liability”) | Environmental Racism: Systemic municipal neglect stemming from historical redlining and white flight.  (“racialized social processes including redlining, blockbusting, white flight and disinvestment”) | Execution of Community Land Trusts (CLTs); Adoption of healing the soil as a tool for racial justice. | **Core Finding:** Farmers navigate the financial trauma of toxic soil remediation while actively resisting environmental racism.  *"Our agricultural system is built on stolen land and stolen labor... If you aren’t willing to have this conversation, you aren’t working for justice."* |
| Silva et al. (2018) [32] | Estonia / Portugal | Roma minorities, migrants | Quantitative GIS analysis | Public Green Spaces | Peripheral Isolation: Relegation to decayed industrial zones completely severed from green services.  (“live in social housing estate neighbourhoods... in abandoned old houses or containers”) | Eco-gentrification: Rent increases driven by high-quality greening, forcing displacement.  (“the cost of housing can rise, and residents may not be able to afford the rent... displacement”) | Deep-seated Segregation: Racial and class-based spatial exclusion of minorities.  (“Roma communities... with low access to public green spaces”) | Deployment of just green enough anti-displacement strategies; Rehabilitation of abandoned private yards for UA. | **Core Finding:** European urban greening often triggers eco-gentrification that expels the vulnerable groups it claims to heal.  *"Green gentrification as the process of displacement or exclusion of the economically most-vulnerable classes of society."* |
| Threlfall et al. (2022) [50] | Melbourne, Australia | Renters, non-English speakers | Spatial analysis; HLM | Street trees, urban forest | Spatial Inequality of Canopy: Significantly lower tree density and viable planting space in disadvantaged zones.  (“disadvantaged groups have disproportionally less access to urban vegetation... unequal spatial distribution”) | Tenancy Constraints: Rental status operating as a primary structural barrier to individual greening actions.  (“income was negatively related to both tree density and diversity... renting... were negatively related”) | Procedural Inequity: Internal municipal biases and linguistic barriers excluding marginalized residents.  (“procedural inequity in management decision-making... English proficiency (negatively) were significant”) | Mandating municipalities to overcome internal biases; Implementation of inclusive incentives for renters. | **Core Finding:** Street tree inequity is driven by systemic municipal bias and the procedural exclusion of renters and minorities.  *"To counteract inequity, local municipalities must acknowledge and deliberately overcome internal biases that favour the inequitable provision of public goods."* |
| Truong et al. (2022) [36] | Sydney, Australia | Social housing tenants | Focus groups | Community gardens | Spatial Marginalization: Relegation of gardens to cramped interstitial spaces like back alleys and parking spots.  (“garden beds were often constructed in the only available spaces... installed in a back alleyway”) | Hidden Economic Burdens: Individual financial precarity in personally funding seeds and tools.  (“participants invested their personal funds... disadvantages in accessing material resources”) | Social Stigmatization: Severe stereotypes causing profound social isolation.  (“stereotypes and prejudices... We're not all junkies, we're not all, you know, mad.”) | Mandating green infrastructure in early social housing planning stages; Utilizing gardens for destigmatizing placemaking. | **Core Finding:** Community gardens act as vital spaces for challenging the dehumanizing stereotypes imposed on the urban poor.  *"The change here turns stereotypes on their head. We’re not all junkies, we’re not all mad. We’re a valued part of the community."* |
| Wardle et al. (2024) [1] | Aberdeen, UK | Vulnerable groups | Semi-structured interviews | Community Gardens | Procedural Complexity: Burdensome logistical processes required to identify owners and secure land rights.  (“complex processes of obtaining land use permissions... finding the landowner, obtaining land rights”) | Grant-seeking Cycles: Absolute reliance on competitive, highly restrictive, theme-based funding.  (“coordinators appeared to be in a continuous cycle of grant seeking”) | Coordinator Burnout: Invisible exploitation and fatigue of unpaid volunteer managers.  (“fatigue or 'taking on too much' as a major challenge... vulnerable groups hit harder by cessation”) | Institutionalization of long-term municipal budgets; Creation of centralized bodies to compensate coordinators. | **Core Finding:** The survival of therapeutic spaces relies precariously on the invisible exploitation of burned-out volunteer labor.  *"The demanding nature of the commitment required... can have negative effects on their wellbeing."* |
| White and Bunn (2017) [43] | Glasgow, UK | Unemployed, activists | Exploratory case study | Community Gardens | Spatial Precarity: Absence of permanent tenure subjecting gardens to unannounced demolition.  (“permanence... is usually at the 'mercy of the landowner'... garden was removed without notice”) | Bureaucratic Exhaustion: Demoralization caused by patchwork funding and tedious grant cycles.  (“demoralised by the 'patchwork' nature... the bureaucracy, the every four years funding cycle”) | The Interim Use Fallacy: Deliberate governmental devaluation of UA as a temporary stopgap.  (“officials typically refer to community gardening as an 'interim use'... stopgap measure”) | Establishment of Food Policy Councils; Utilization of Community Empowerment Acts for collective land acquisition. | **Core Finding:** Governments weaponize the interim use designation to treat gardens as vulnerable placeholders for capital.  *"UA remains a grassroots movement that operates... 'in spite of planning'."* |
| Witheridge and Morris (2016) [18] | Edinburgh, UK | Socially isolated groups | Policy analysis; Interviews | Community Gardens | Rigid Mono-zoning: Bureaucratic mapping masking potential interstitial spaces suitable for cultivation.  (“mono-zoning... makes it difficult to identify other areas... for cultivation”) | Mission Creep: Neoliberal reliance on short-term competitive grants forcing deviation from core therapeutic goals.  (“funding structure can result in mission creep... hinder mid- to long-term viability”) | Neoliberal Marginalization: Framing of gardens as mere apolitical alternatives without collective rights.  (“neoliberalist policies create an environment in which 'alternative' urban development... is no longer viable”) | Adoption of reasoning backwards planning; Strategic prominent localization to increase political visibility. | **Core Finding:** Short-term neoliberal grants coerce grassroots gardens into exhausting cycles of mission creep.  *"It threatens to curtail the 'politics of possibility' for a local food system."* |
| Wolch et al. (2014) [9] | USA and China | Low-income, racial minorities | Literature review | Urban Parks and Greenways | Park Poverty: Severe lack of green space and disproportionate exposure to environmental hazards.  (“park-adjacent neighborhoods... had higher pollution... especially in low income and minority communities”) | Spending Inequity: Vastly lower municipal and non-profit expenditure directed toward marginalized neighborhoods.  (“low-income communities of color having far less to spend on parks and recreation”) | Eco-Gentrification: The Green Paradox where ecological makeovers directly displace the poor.  (“housing costs can rise... potentially lead to gentrification: the displacement and/or exclusion”) | Implementation of just green enough strategies integrating scattered sites with rent stabilization. | **Core Finding:** High-capital greening projects act as tools of displacement, creating a violent green paradox.  *"Redressing park-poverty... can create an urban green space paradox... potentially leading to gentrification."* |
| Zhang et al. (2025) [6] | Jinan, China | Elderly population | Spatial regression models | Urban green spaces | Spatial Fragmentation: High-intensity land development and built-area encroachment destroying natural spaces.  (“urbanization pressures and the rapid expansion of built-up areas have often led to the encroachment”) | The Luxury Effect: Prioritization of economic growth over equitable green space provision in older areas.  (“the 'luxury effect', where wealthier neighborhoods tend to have more green space due to greater financial resources”) | Planning Legacy Effects: Institutional bias favoring new facility construction over preservation.  (“the 'legacy effect'... older developments may lack the green infrastructure... schools and hospitals often prioritize the construction of new facilities”) | Integration of green infrastructure into core planning; Legislation protecting green spaces in public institutions. | **Core Finding:** Green space allocation is trapped by the luxury effect and legacy effect, marginalizing older districts.  *"Socio-economic conditions—such as wealth level, planning legacy, and functional land designation—are pivotal."* |
| Zhang et al. (2021) [49] | Beijing, China | Vulnerable groups | Spatial visualization | Community green spaces | Historical Spatial Limitations: Extremely scattered spaces in traditional, dense urban cores.  (“communities within the 2nd ring... have extremely limited and scattered available green space areas”) | Class-based Displacement: Strict correlation between green space allocation and socioeconomic status.  (“related to socioeconomic status of residents... the housing displacement of vulnerable groups”) | Deep Social Segregation: Spatial inequity manifesting strictly as an exclusionary residential class issue.  (“Social segregation exists in the use of green space... makes green space equity more represents as residential problems.”) | Integration of fine-scale morphological indicators and scientific evaluation frameworks in old residential retrofitting. | **Core Finding:** High-density historical cores exacerbate green space fragmentation, intensifying class-based residential segregation.  *"Social isolation is manifested mainly in uneven access to green space, the housing displacement of vulnerable groups... which makes green space equity more represents as residential problems."* |
|  |  |  |  |  |  |  |  |  |  |

**Appendix C**

**Analytical Framework and Exclusion Rationale**

**C.1.Rationale for Exclusion at Full-Text Screening (n=43)**

Full-text exclusions were strictly executed based on the predetermined Population, Concept, and Context (PCC) framework. Studies failing to explicitly address socio-spatial structural barriers were systematically eliminated. Table C1 details representative exclusions and their corresponding methodological rationales.

**Table C1.** Representative List of Excluded Studies and Methodological Rationales.

| **Author (Year)** | **Journal** | **Primary Exclusion Category** | **Detailed Methodological Rationale** |
| --- | --- | --- | --- |
| Bagstad and Shammin (2012) [S1] | Ecological Indicators | Macroeconomic Indicator Focus | Excluded due to macroeconomic focus (Genuine Progress Indicator) rather than structural socio-spatial barrier analysis. |
| Baybutt et al. (2019) [S2] | Health Promotion International | Contextual Deviation (Prisons) | Excluded due to institutional context deviation (prison settings) incompatible with urban spatial planning parameters. |
| Fox (2022) [S3] | Entropy | Biosocial-Technical Focus | Excluded due to digital/theoretical focus (digital twins) lacking empirical evaluation of urban structural barriers. |
| García-Llorente et al. (2018) [S4] | IJERPH | Conceptual Deviation (Green Care) | Excluded due to conceptual focus on clinical effectiveness of therapeutic farming, devoid of spatial or institutional barrier assessment. |
| Haluza et al. (2025) [21] | Sustainability | Study Design (Bibliometric) | Excluded due to study design (bibliometric analysis) lacking original empirical data extraction on socio-spatial barriers. |
| Kingsley et al. (2019) [S5] | IJERPH | Publication Type (Protocol) | Excluded due to publication type (study protocol) lacking finalized empirical barrier data. |
| Koay and Dillon (2020) [S6] | IJERPH | Health Outcomes Exclusivity | Excluded due to exclusive focus on quantitative health outcomes, lacking analysis of Physical and Spatial Barriers or Institutional and Social Exclusion. |
| Orta-Ortiz and Geneletti (2023) [S7] | Env. Impact Assessment Review | Biophysical Modeling Focus | Excluded due to biophysical efficiency modeling (Nature-based Solutions) overriding the evaluation of Economic and Financial Barriers. |
| Tracey et al. (2020) [S8] | IJERPH | Publication Type (Protocol) | Excluded due to publication type (systematic review protocol) devoid of empirical structural barrier synthesis. |
| Tuominen et al. (2022) [S9] | Scientific Reports | Internal Group Dynamics Focus | Excluded due to focus on internal group behavioral dynamics rather than systemic Institutional and Social Exclusion. |

**C.2. Thematic Analysis Codebook**

The thematic synthesis was conducted via Reflexive Thematic Analysis [29,30]. Table C2 maps the conceptual transition from raw extracted indicators to the overarching thematic framework governing this scoping review.

**Table C2.** Coding Structure for the Triad of Structural Barriers.

| **Overarching Theme** | **Sub-themes (Secondary Codes)** | **Initial Codes (Raw Indicators) [Source Examples]** |
| --- | --- | --- |
| 1. Physical and Spatial Barriers | Land Tenure Precarity; Microclimate Hostility; Spatial Fragmentation | Temporary permits and bulldozing risks [31,42]; High-density crowding and spatial fragmentation [45,49]; Microclimatic and wind hostility [47]. |
| 2. Economic and Financial Barriers | Eco-gentrification Threats; Neoliberal Funding Dependencies; Resource Disparities | Green gentrification and displacement threats [32,46]; Exploitation of unpaid volunteer labor [36,51]; The luxury effect and capital disparities [6,50]. |
| 3. Institutional and Social Exclusion | Procedural Inequity; Systemic Stigmatization; Epistemic Violence | Top-down planning silos and bureaucratic red tape [13,34]; Stigmatization of marginalized bodies [36,52]; Racial bias and marginalization of indigenous knowledge [10,17]. |

**Supplementary References**

S1. Bagstad, K.J.; Shammin, M.R. Can the Genuine Progress Indicator better inform sustainable regional progress? —A case study for Northeast Ohio. *Ecol. Indic.* **2012**, *18*, 330–341. https://doi.org/10.1016/j.ecolind.2011.11.026
S2. Baybutt, M.; Dooris, M.; Farrier, A. Growing health in UK prison settings. *Health Promot. Int.* **2019**, *34*, 792–802. https://doi.org/10.1093/heapro/day037
S3. Fox, S. Synchronous generative development amidst situated entropy. *Entropy* **2022**, *24*, 89. https://doi.org/10.3390/e24010089
S4. García-Llorente, M.; Rubio-Olivar, R.; Gutierrez-Briceño, I. Farming for life quality and sustainability: A literature review of green care research trends in Europe. *Int. J. Environ. Res. Public Health* **2018**, *15*, 1282. https://doi.org/10.3390/ijerph15061282
S5. Kingsley, J.; Bailey, A.; Torabi, N.; Zardo, P.; Mavoa, S.; Gray, T.; et al. A systematic review protocol investigating community gardening impact measures. *Int. J. Environ. Res. Public Health* **2019**, *16*, 3430. https://doi.org/10.3390/ijerph16183430
S6. Koay, W.I.; Dillon, D. Community gardening: Stress, well-being, and resilience potentials. *Int. J. Environ. Res. Public Health* **2020**, *17*, 6740. https://doi.org/10.3390/ijerph17186740
S7. Orta-Ortiz, M.S.; Geneletti, D. Prioritizing urban nature-based solutions to support scaling-out strategies: A case study in Las Palmas de Gran Canaria. *Environ. Impact Assess. Rev.* **2023**, *102*, 107158. https://doi.org/10.1016/j.eiar.2023.107158
S8. Tracey, D.; Gray, T.; Sweeting, J.; Kingsley, J.; Bailey, A.; et al. A systematic review protocol to identify the key benefits and associated program characteristics of community gardening for vulnerable populations. *Int. J. Environ. Res. Public Health* **2020**, *17*, 2029. https://doi.org/10.3390/ijerph17062029
S9. Tuominen, L.S.; Helle, S.; Helanterä, H.; Karell, P.; Rapeli, L.; et al. Structural equation modeling reveals decoupling of ecological and self-perceived outcomes in a garden box social-ecological system. *Sci. Rep*. **2022**, *12*, 10178. https://doi.org/10.1038/s41598-022-10178-z
